# Metabolic Risk Signals in Periodontal Clinics: Cross-Sectional Associations of PISA with hs-CRP and HOMA-IR

**DOI:** 10.3390/diagnostics16131972

**Published:** 2026-06-25

**Authors:** Maria-Alexandra Martu, Ioana Martu, Sorina Mihaela Solomon, Ionut Luchian, Silvia Martu, Liliana Pasarin, Diana-Maria Anton, Monica Mihaela Scutariu, Cezar-Ilie Foia, Elena-Odette Luca, Irina-Georgeta Sufaru

**Affiliations:** Grigore T. Popa University of Medicine and Pharmacy, 700115 Iasi, Romania; maria-alexandra.martu@umfiasi.ro (M.-A.M.); sorina.solomon@umfiasi.ro (S.M.S.); ionut.luchian@umfiasi.ro (I.L.); silvia.martu@umfiasi.ro (S.M.); liliana.pasarin@umfiasi.ro (L.P.); diana_maria_a@yahoo.com (D.-M.A.); foia.cezar-ilie@d.umfiasi.ro (C.-I.F.); elena-odette.luca@umfiasi.ro (E.-O.L.); ursarescu.irina@umfiasi.ro (I.-G.S.)

**Keywords:** high-sensitivity C-reactive protein (hs-CRP), insulin resistance, HOMA-IR, periodontal inflamed surface area (PISA), periodontitis, systemic inflammation

## Abstract

**Background/Objectives**: Periodontitis can elevate systemic inflammation and negatively impact glucose regulation. This cross-sectional study investigated relationships between periodontal inflammatory burden, measured by periodontal inflamed surface area (PISA), and insulin resistance and systemic inflammation in never-smokers attending a periodontal clinic with unknown diabetes status. **Methods**: A total of 154 adults underwent full-mouth periodontal assessments to determine PISA. Fasting blood samples were taken for glucose, insulin, and hs-CRP, and HOMA-IR was calculated. An inflammation composite was created from z-scored log(hs-CRP), IL-6, and TNF-α. Primary analysis involved regressing log(HOMA-IR) and log(hs-CRP) on PISA, scaled by interquartile range (IQR) increases, with sequential adjustments. Mediation analysis, adjusted for age, sex, and waist circumference, used bootstrap testing to evaluate inflammation as a mediator. **Results**: HOMA-IR and hs-CRP increased across PISA tertiles (HOMA-IR: 2.01 to 3.17; hs-CRP: 0.73 to 2.48 mg/L; both *p* < 0.001). In the prespecified primary adjusted model (age, sex, waist circumference, education, physical activity, family history of diabetes, and medication use), each IQR increase in log(PISA) was associated with 85.2% higher hs-CRP (95% CI 53.1% to 124.1%; *p* < 0.001) and 15.7% higher HOMA-IR (95% CI −0.8% to 34.9%; *p* = 0.064). Estimates were similar with additional adjustment for BMI and SBP, whereas adding plaque (% sites) attenuated associations (hs-CRP: +15.2%; *p* = 0.234; HOMA-IR: +1.0%; *p* = 0.925). Mediation analysis (adjusted for age/sex/waist circumference) was consistent with an indirect pathway via the inflammation composite (a × b = 0.212; 95% CI 0.094 to 0.337), while the direct effect was not supported (c′ = −0.047; 95% CI −0.207 to 0.125). **Conclusions**: Higher periodontal inflammatory burden was strongly associated with systemic inflammation and showed weaker, model-dependent associations with insulin resistance; the findings were consistent with an inflammation-linked pathway in exploratory, partially adjusted mediation analyses; given the cross-sectional design, causal inference is not possible.

## 1. Introduction

Periodontitis is a common, chronic inflammatory disease driven by dysbiotic biofilms and sustained by an imbalanced host response, resulting in the gradual loss of tissues that support the teeth [1,2]. Beyond local effects, recent research increasingly links periodontitis to systemic inflammation through repeated exposure of ulcerated epithelium, the presence of bacteria or their toxins in the bloodstream, and the spread of inflammatory molecules [3,4,5]. These mechanisms are particularly significant for metabolic health, as low-grade systemic inflammation is known to disrupt insulin function and increase cardiometabolic risk [6,7,8].

Diabetes mellitus and periodontitis are linked by a well-established bidirectional relationship. Hyperglycemia and insulin resistance can exacerbate periodontal inflammation by impairing neutrophil function, amplifying cytokine responses, impairing wound healing, and disrupting microvascular health [7,9]. Conversely, periodontitis can negatively affect blood sugar control by increasing systemic inflammation. Recent reviews have supported the clinical and mechanistic understanding of this connection, emphasizing inflammation as a key linking factor [10,11]. However, in observational studies, the strength of these associations varies widely due to differences in periodontal classification, population risk factors, and the extent to which shared influences such as age, adiposity, and socioeconomic and behavioral factors are controlled [12].

A key challenge in this field is measuring periodontal “exposure” in a way that captures active inflammation rather than relying solely on categorical case definitions. The periodontal inflamed surface area (PISA) was developed to estimate the bleeding pocket epithelial surface, thereby operationalizing the concept of an “inflammatory wound” in the periodontium [13]. This quantitative measure has been associated with systemic inflammatory biomarkers in cross-sectional studies, supporting its utility as an exposure indicator for systemic outcomes [14,15].

Regarding metabolic outcomes, insulin resistance is an important intermediate phenotype, reflecting an early, potentially modifiable stage in progression to dysglycemia [16]. The Homeostasis Model Assessment of Insulin Resistance (HOMA-IR), calculated from fasting insulin and glucose levels, remains widely used in epidemiologic research as a practical indicator of insulin resistance [17,18]. Recent population studies have linked insulin resistance markers (including HOMA-IR) to periodontitis, though these associations are attenuated after adjustment for adiposity and other shared risk factors [12]. Studies that include both PISA and insulin resistance measures further support a possible link between periodontal and metabolic health, yet they also highlight variability across study designs and populations [19].

Systemic inflammation is a complementary and clinically meaningful target for research linking periodontal and metabolic health. High-sensitivity C-reactive protein (hs-CRP) is a reliable, standardized marker of low-grade systemic inflammation [20] and has been consistently associated with measures of periodontal inflammation [21]. Recent data-driven studies have also confirmed that the inflammatory burden from periodontal disease significantly contributes to systemic inflammation, even after accounting for other systemic factors [15].

From a translational perspective, dental settings have been proposed as potential venues for identifying individuals at elevated cardiometabolic risk, given the common co-occurrence of periodontal disease, obesity, and dysglycemia. However, the practical utility of such an approach remains uncertain, and research on diabetes risk screening in dental clinics has highlighted substantial barriers as well as opportunities for implementation [22,23]. In this context, a clearer characterization of how periodontal inflammatory burden relates to systemic inflammation and insulin resistance—particularly in well-controlled populations limiting confounding by tobacco use—could inform future research on metabolic risk stratification, while recognizing that clinical translation would require prospective validation in broader settings.

Despite growing interest in PISA as a quantitative measure of exposure, most prior studies either use categorical periodontal definitions, include smokers, or focus on populations with known or pre-screened diabetes status—factors that may obscure the true relationship between active periodontal inflammation and metabolic outcomes. To our knowledge, few studies have simultaneously examined PISA in relation to both insulin resistance and systemic inflammation while restricting the sample to never-smokers with unknown diabetes status at enrollment, or have formally tested whether systemic inflammation mediates the PISA–insulin resistance relationship using a composite inflammatory index.

We therefore hypothesized that higher periodontal inflammatory burden, measured continuously by PISA, would be positively associated with systemic inflammation (hs-CRP) and insulin resistance (HOMA-IR) in never-smokers attending a periodontal clinic with previously unknown diabetic status, and that systemic inflammation—operationalized as a composite of hs-CRP, IL-6, and TNF-α—would partially mediate the association between PISA and HOMA-IR. Secondary objectives were to characterize dose–response patterns across PISA tertiles and to evaluate the robustness of associations through sequential sensitivity analyses.

## 2. Materials and Methods

### 2.1. Study Design, Setting, and Reporting

This cross-sectional observational study was conducted at the Periodontology Clinic of Grigore T. Popa University of Medicine and Pharmacy in Romania. The research examined the association between periodontal inflammatory burden and (i) insulin resistance, measured using the homeostatic model assessment (HOMA-IR), and (ii) systemic inflammation, assessed by high-sensitivity C-reactive protein (hs-CRP) levels and a predefined inflammation composite score. The study adhered to the STROBE (Strengthening the Reporting of Observational Studies in Epidemiology) guidelines.

All procedures were conducted in accordance with the Declaration of Helsinki and applicable local regulations. The study protocol was approved by the Institutional Review Ethics Committee of Grigore T. Popa University of Medicine and Pharmacy in Iasi, Romania (approval date: 30 July 2020). All participants provided written informed consent before enrollment. The data were coded, securely stored on password-protected systems, and managed in compliance with applicable data protection laws.

### 2.2. Participants

Eligible participants were adults (≥18 years) who reported never smoking. Their diabetes status was unknown at enrollment. The following exclusion criteria were applied: a history of physician-diagnosed diabetes mellitus, current or past use of glucose-lowering medications, or a history of gestational diabetes. Additional exclusions included pregnancy or lactation; a recent acute systemic infection within the past 2 weeks; use of systemic antibiotics within the last 3 months; chronic inflammatory or autoimmune conditions requiring immunosuppressive therapy; and health issues that affect HbA1c interpretation, such as hemoglobinopathies or recent blood transfusions, as known from medical history.

**Figure 1:**
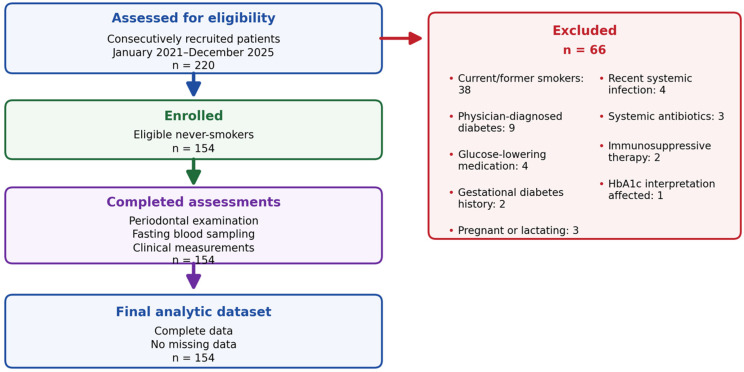
The participant flow diagram.

Participants were consecutively recruited from January 2021 to December 2025. Of 220 patients initially assessed for eligibility, 66 were excluded: 38 were current or former smokers; 9 had a physician-diagnosed history of diabetes mellitus; 4 were using glucose-lowering medications; 2 reported a history of gestational diabetes; 3 were pregnant or lactating; 4 had a recent acute systemic infection within the preceding 2 weeks; 3 had used systemic antibiotics within the last 3 months; 2 had chronic inflammatory or autoimmune conditions requiring immunosuppressive therapy; and 1 had conditions affecting HbA1c interpretation. All 154 enrolled participants completed the study assessments, yielding a complete dataset with no missing data. A participant flow diagram is provided in Figure 1.

### 2.3. Periodontal Assessment and PISA Calculation

All periodontal examinations were performed by a single calibrated periodontist using standardized procedures. Full-mouth periodontal charting was recorded at six sites per tooth (mesiobuccal, mid-buccal, distobuccal, mesiolingual, mid-lingual, distolingual), excluding third molars. Probing pocket depth (PPD) and clinical attachment level (CAL) were measured to the nearest millimeter with a UNC-15 periodontal probe (Hu-Friedy, Chicago, IL, USA) using a light, standardized probing force (approximately 0.25 N). Bleeding on probing (BOP) was recorded as present or absent within 10 s after probing [24]. Plaque was assessed using a dichotomous plaque index (presence/absence) at four sites per tooth. Plaque percentage was calculated as the proportion of assessed sites with visible plaque (presence/absence), multiplied by 100. Tooth mobility and furcation involvement were recorded when present.

Prior to participant recruitment, the examining periodontist underwent a structured calibration exercise under the supervision of an experienced senior periodontist. Calibration comprised two dedicated training sessions conducted on volunteer patients not included in the main study, during which the examiner performed full-mouth periodontal charting alongside the supervising periodontist; discrepancies in PPD and CAL recordings were reviewed, discussed, and resolved before data collection commenced. A minimum intraclass correlation coefficient (ICC) of 0.80 was pre-specified as the threshold for acceptable reliability. Intra-examiner reliability was subsequently assessed on a convenience sample of 15 periodontitis patients attending the clinic (not included in the main analysis), yielding approximately 2340 re-examined sites for each of PPD and CAL (15 subjects × mean 26 teeth × 6 sites per tooth). The re-examination was performed after an interval of 7–10 days, with the examiner blinded to the first set of recordings, using the same standardized probing force (approximately 0.25 N) and UNC-15 probe as in the main study. Intra-examiner reliability exceeded the pre-specified threshold for both PPD (ICC = 0.92) and CAL (ICC = 0.89), and 90.0% of repeated measurements were within ±1 mm of the original value, consistent with acceptable calibration for epidemiological periodontal research.

The periodontal inflamed surface area (PISA) was used as the primary measure of periodontal exposure, representing the surface area of bleeding pocket epithelium (mm^2^). PISA was calculated at each site using PPD, CAL (to estimate the periodontal epithelial surface), and BOP status, following established algorithms [25]. Higher PISA values indicated a greater periodontal inflammatory burden. For descriptive purposes, participants were divided into three PISA groups (tertiles).

Periodontitis was classified according to the 2018 AAP/EFP classification for periodontitis staging and grading [26].

### 2.4. Anthropometric and Clinical Measurements

Height was measured without shoes using a wall-mounted stadiometer (seca 217, seca GmbH & Co. KG, Hamburg, Germany), and body weight was measured in light clothing with a calibrated digital scale (seca 877, seca GmbH & Co. KG, Hamburg, Germany). Body mass index (BMI) was calculated as weight (kg) divided by height (m) squared. Waist circumference was measured at the midpoint between the lowest rib and the iliac crest with a non-elastic measuring tape (seca 201, seca GmbH & Co. KG, Hamburg, Germany). Resting blood pressure was measured after at least 5 min of seated rest with an automated oscillometric monitor (OMRON HEM-907, Omron Healthcare, Kyoto, Japan). Three readings were taken 1 min apart, and the average of the last 2 was used for analysis.

### 2.5. Metabolic and Inflammatory Biomarkers

Venous blood was collected in the morning after an overnight fast of at least 8 h. Samples were drawn into serum separator and EDTA tubes (BD Vacutainer, Becton, Dickinson and Company, Franklin Lakes, NJ, USA). Serum and plasma were separated by centrifugation at 1500× *g* for 10 min at 4 °C using a refrigerated centrifuge (Eppendorf 5702 R, Eppendorf AG, Hamburg, Germany) within 60 min of collection. Aliquots were stored at −80 °C in an ultra-low-temperature freezer (Thermo Scientific Forma 900 Series, Thermo Fisher Scientific, Waltham, MA, USA) until analysis.

Fasting plasma glucose was measured by the hexokinase method on an automated chemistry analyzer (cobas c 501, Roche Diagnostics, Rotkreuz, Switzerland). Fasting serum insulin was quantified by electrochemiluminescence immunoassay (ECLIA) on an automated immunoassay analyzer (cobas e 411, Roche Diagnostics, Rotkreuz, Switzerland).

HbA1c was measured in EDTA whole blood by high-performance liquid chromatography (HPLC) using an HbA1c analyzer (D-100, Bio-Rad Laboratories, Hercules, CA, USA) and was traceable to IFCC/NGSP standards. HbA1c was measured for descriptive characterization of glycemic status in the study cohort and to support post-assessment glycemic categorization.

High-sensitivity C-reactive protein (hs-CRP) was measured by particle-enhanced immunoturbidimetry on the cobas c 501 system (Roche Diagnostics, Rotkreuz, Switzerland). All assays followed manufacturers’ instructions, with daily internal quality control procedures and, where applicable, external proficiency testing. Interleukin-6 (IL-6) and tumor necrosis factor-alpha (TNF-α) were measured in serum using high-sensitivity ELISA kits (R&D Systems, Minneapolis, MN, USA). Absorbance was read on a microplate reader (Synergy H1, BioTek Instruments, Winooski, VT, USA), and concentrations were calculated from 4-parameter logistic standard curves using Gen5 software (version 3.16; BioTek Instruments, Winooski, VT, USA). Assay sensitivity, ranges, and intra- and inter-assay coefficients of variation followed manufacturers’ specifications.

### 2.6. Outcomes

The primary metabolic measure was insulin resistance, assessed by HOMA-IR, calculated as fasting insulin (µU/mL) × fasting glucose (mmol/L)/22.5. If glucose was recorded in mg/dL, it was converted to mmol/L by dividing by 18.0. The main inflammatory marker was hs-CRP (mg/L).

Additionally, a predefined inflammation composite score was computed to serve as a multidimensional mediator in the mediation analysis. The three markers were selected because they capture complementary and mechanistically relevant aspects of the systemic inflammatory response: TNF-α and IL-6 are upstream pro-inflammatory cytokines directly implicated in periodontal-systemic crosstalk and in the disruption of insulin signaling, whereas hs-CRP is a sensitive downstream acute-phase reactant that integrates upstream cytokine activity, particularly IL-6 signaling, and provides a stable, standardized measure of low-grade systemic inflammation. Each marker was log-transformed to address right skew and then standardized (z-scored) to place all three on a common unit-free scale, thereby preventing any single marker from dominating the composite by virtue of its measurement scale or variance. The composite was calculated as the unweighted mean of the three z-scores. Equal weighting was chosen a priori as the most transparent and reproducible approach and to avoid data-driven weight estimation, which can capitalize on sample-specific variance structure and reduce generalizability in samples of this size. As a post hoc check, principal component analysis (PCA) of the log-transformed, standardized markers confirmed that PC1, which explained 57.9% of total variance, had nearly identical loadings for hs-CRP (0.594), IL-6 (0.566), and TNF-α (0.572), and that the equal-weight composite was virtually identical to the PC1 score (r = 1.00). Moderate inter-marker correlations on the log scale (*r* = 0.34–0.39) and a Cronbach’s α of 0.62 indicated that the three markers shared a common inflammatory signal while remaining sufficiently distinct to justify their combined use.

HbA1c was treated as a secondary descriptive variable and was not used as a primary endpoint.

### 2.7. Data Management and Quality Assurance

Clinical data were collected using standardized case report forms and stored in a password-protected electronic database. Range and logic checks were performed on key variables. The examining periodontist was calibrated before recruitment began, as described in Section 2.3. Laboratory analyses were performed by personnel blinded to the periodontal measurements.

### 2.8. Statistical Analysis

Analyses were performed in R (R Foundation for Statistical Computing, Vienna, Austria). Continuous variables were summarized as means (standard deviations) or medians (interquartile ranges), and categorical variables as counts (percentages). Skewed variables, including hs-CRP, IL-6, TNF-α, fasting insulin, and HOMA-IR, were log-transformed prior to modeling. Periodontal inflammatory burden (PISA) was log-transformed and scaled such that regression coefficients corresponded to an interquartile range (IQR) increase in log(PISA). Across-tertile comparisons used one-way analysis of variance (ANOVA) for continuous variables summarized as mean (SD), Kruskal–Wallis tests for continuous variables summarized as median [IQR], and chi-square tests for categorical variables.

Primary associations were evaluated using multivariable linear regression with log(HOMA-IR) and log(hs-CRP) as dependent variables and log(PISA) as the main independent variable. The prespecified primary adjusted model included age, sex, waist circumference, education (years), physical activity category, family history of diabetes, and medication use (statins, antihypertensives, systemic corticosteroids). Sensitivity models were additionally adjusted for BMI, systolic blood pressure (SBP), plaque (% sites), and a combined model including both BMI and plaque. Model results are presented as percent change in the outcome per IQR increase in log(PISA), with 95% confidence intervals (CIs). Robust (HC3) standard errors were used.

**Figure 2:**
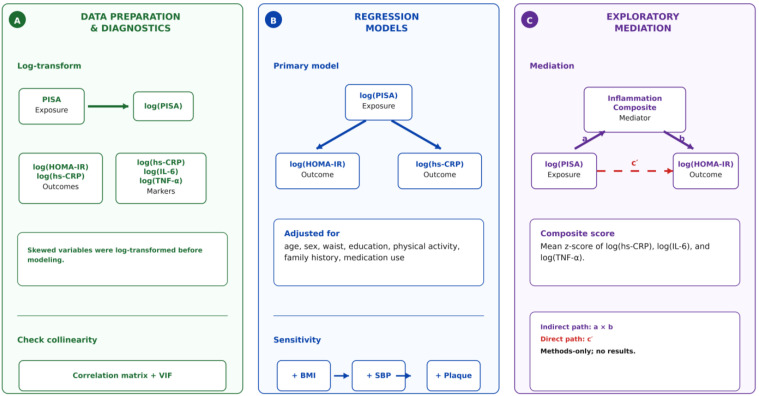
Overview of the statistical analysis framework. The diagram summarizes the five analytical steps applied in the study, their roles, and their relationships to the primary exposure (PISA), covariates, and outcomes (HOMA-IR and hs-CRP). Solid blue arrows indicate primary regression pathways; the dashed red pathway indicates the exploratory mediation analysis. All skewed variables were log-transformed prior to modeling; regression results are expressed as percentage change per interquartile range (IQR) increase in log(PISA). PISA: periodontal inflamed surface area; HOMA-IR: Homeostasis Model Assessment of Insulin Resistance; hs-CRP: high-sensitivity C-reactive protein; SBP: systolic blood pressure.

Potential non-linearity was assessed using restricted cubic splines for log(PISA) (4 degrees of freedom) and compared with the linear specification using likelihood ratio tests. Multicollinearity was assessed using variance inflation factors (VIF). Effect modification by central adiposity was explored by testing an interaction term between log(PISA) and waist circumference. Influence diagnostics were evaluated using Cook’s distance (threshold 4/n). A two-sided *p*-value < 0.05 was considered statistically significant. The main statistical methods applied are presented in Figure 2.

### 2.9. Sample Size Considerations

The target sample size of approximately 150 participants was set in advance to ensure sufficient precision for continuous outcomes and to detect moderate relationships between periodontal inflammatory burden and metabolic or inflammatory biomarkers. With this sample size, the study had about 80% power (two-sided α = 0.05) to detect a Pearson correlation of roughly 0.23 between PISA and continuous outcomes, as well as standardized mean differences of about 0.45 between the highest and lowest PISA tertiles, assuming balanced group sizes.

## 3. Results

### 3.1. Participant Characteristics

All 154 participants completed the study assessments, yielding complete datasets with no missing data. None of the participants were smokers. The cohort (N = 154) was evenly distributed across PISA tertiles (T1: n = 51; T2: n = 51; T3: n = 52). A higher periodontal inflammatory burden was associated with less favorable anthropometric and periodontal profiles. Participants in the highest tertile (T3) were older than those in T1 (52.1 ± 12.0 vs. 46.2 ± 12.5 years; *p* = 0.035) and had greater adiposity, including higher BMI (28.7 ± 4.3 vs. 26.9 ± 4.0 kg/m^2^; *p* = 0.010) and larger waist circumference (99.5 ± 12.4 vs. 89.8 ± 16.1 cm; *p* = 0.002). Blood pressure did not differ significantly across tertiles (SBP: *p* = 0.351; DBP: *p* = 0.083) (Table 1).

As expected, periodontal parameters showed clear gradients across tertiles: compared with T1, T3 had fewer teeth (24.3 ± 2.1 vs. 27.4 ± 1.2; *p* < 0.001), higher mean probing depth (3.9 ± 0.8 vs. 2.8 ± 0.7 mm; *p* < 0.001), and greater clinical attachment loss (3.2 ± 1.2 vs. 1.6 ± 1.1 mm; *p* < 0.001). Bleeding on probing was also notably higher (68.2 ± 16.4% vs. 26.6 ± 16.4%; *p* < 0.001), as were plaque levels (62.9 ± 16.8% vs. 24.9 ± 15.7%; *p* < 0.001) (Table 2).

Median PISA increased from 225.0 [157.0–264.5] mm^2^ in T1 to 1092.5 [840.5–1432.8] mm^2^ in T3 (*p* < 0.001) (Table 2). Importantly, biochemical indices varied with periodontal inflammatory burden: median HOMA-IR rose from 2.01 [1.31–3.82] in T1 to 3.17 [2.43–5.63] in T3 (*p* < 0.001), and median hs-CRP increased from 0.73 [0.48–1.01] mg/L to 2.48 [1.36–4.35] mg/L (*p* < 0.001) (Table 3). Fasting glucose, fasting insulin, IL-6, and TNF-α also differed across tertiles, whereas HbA1c showed only a modest gradient that did not reach statistical significance.

Conversely, sex distribution, family history of diabetes, physical activity level, and medication use (statins, antihypertensive medications, systemic corticosteroids) were similar across tertiles (all *p* > 0.05) (Table 1), indicating that the observed gradients primarily reflect differences in periodontal inflammation and adiposity rather than significant imbalances in these covariates.

According to staging/grading, Stage IV (44/154; 28.6%) and Stage I (43/154; 27.9%) were the most frequent, followed by Stage II (36/154; 23.4%) and Stage III (31/154; 20.1%) (Table 4). In terms of grading, Grade A (64/154; 41.6%) and Grade B (56/154; 36.4%) predominated, while Grade C accounted for 34/154 (22.1%) (Table 4).

### 3.2. Primary Outcomes

In the primary adjusted models (age, sex, waist circumference, education, physical activity category, family history of diabetes, and medication use), an IQR increase in log(PISA) was associated with an 85.2% higher hs-CRP (95% CI 53.1% to 124.1%; *p* < 0.001) and a 15.7% higher HOMA-IR (95% CI −0.8% to 34.9%; *p* = 0.064). In sensitivity models, additional adjustment for BMI yielded similar estimates (HOMA-IR: +15.1%; *p* = 0.063; hs-CRP: +83.9%; *p* < 0.001) (Table 5).

Adjustment for SBP did not materially change the hs-CRP association (+85.8%; *p* < 0.001) and resulted in a modest association with HOMA-IR (+17.4%; *p* = 0.034). When plaque (% sites) was added to the primary adjusted model, associations attenuated (HOMA-IR: +1.0%; *p* = 0.925; hs-CRP: +15.2%; *p* = 0.234) (Table 5), consistent with plaque being a proximal determinant of periodontal inflammation and a potential source of overadjustment when estimating total associations with systemic outcomes.

**Figure 3:**
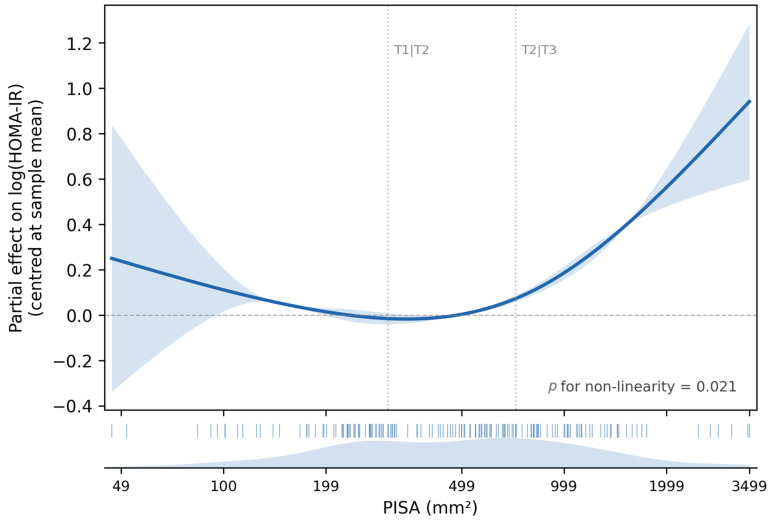
Restricted cubic spline showing the adjusted association between log-transformed periodontal inflamed surface area (PISA) and log-transformed HOMA-IR, in the model adjusted for age, sex, and waist circumference (4 degrees of freedom; *p* for non-linearity = 0.021). The solid line represents the spline fit; the shaded area represents the 95% confidence interval. Individual observations are shown as a rug plot along the *x*-axis, and the overlaid kernel density estimate illustrates the distribution of PISA values across the sample. Vertical dashed lines indicate PISA tertile boundaries (T1/T2 and T2/T3).

**Figure 4:**
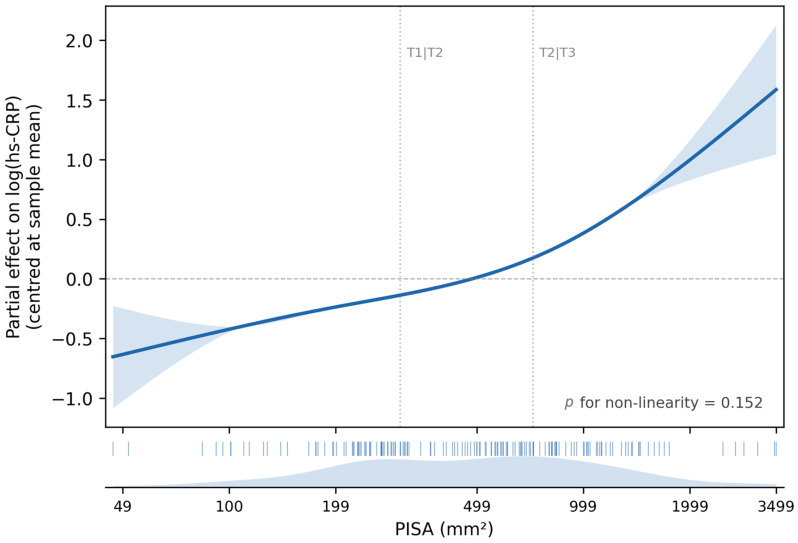
Restricted cubic spline showing the adjusted association between log-transformed periodontal inflamed surface area (PISA) and log-transformed hs-CRP, in the model adjusted for age, sex, and waist circumference (4 degrees of freedom; *p* for non-linearity = 0.152). The solid line represents the spline fit; the shaded area represents the 95% confidence interval. Individual observations are shown as a rug plot along the *x*-axis, and the overlaid kernel density estimate illustrates the distribution of PISA values across the sample. Vertical dashed lines indicate PISA tertile boundaries (T1/T2 and T2/T3).

Variance inflation factors indicated low multicollinearity in the primary adjusted model (all VIFs ≤ 3.9; waist circumference VIF ≈ 1.37). Restricted cubic spline analyses based on models adjusted for age, sex, and waist circumference suggested non-linearity for the association between PISA and HOMA-IR (*p* for non-linearity = 0.021; Figure 3), whereas non-linearity was not supported for the association between PISA and hs-CRP (*p* = 0.152; Figure 4).

Influence diagnostics identified 10 observations exceeding Cook’s distance 4/n in each primary model; exclusion of these observations did not materially alter the hs-CRP association and modestly strengthened the HOMA-IR estimate. No evidence of effect modification by waist circumference was observed (*p* for interaction = 0.844 for HOMA-IR; *p* = 0.179 for hs-CRP). Exclusion of participants with diabetes-range values (n = 7) yielded similar results.

### 3.3. Inflammation Composite and Mediation Analysis

In the pre-specified exploratory, cross-sectional mediation analysis, adjusted for age, sex, and waist circumference (Table 6), the association between periodontal inflammatory burden (PISA; per IQR increase in log-transformed PISA) and insulin resistance (HOMA-IR) was consistent with an indirect pathway via the inflammation composite. The total effect of PISA on HOMA-IR was positive (c = 0.165; 95% CI 0.021 to 0.314), corresponding to an approximately 18.0% increase in HOMA-IR.

**Figure 5:**
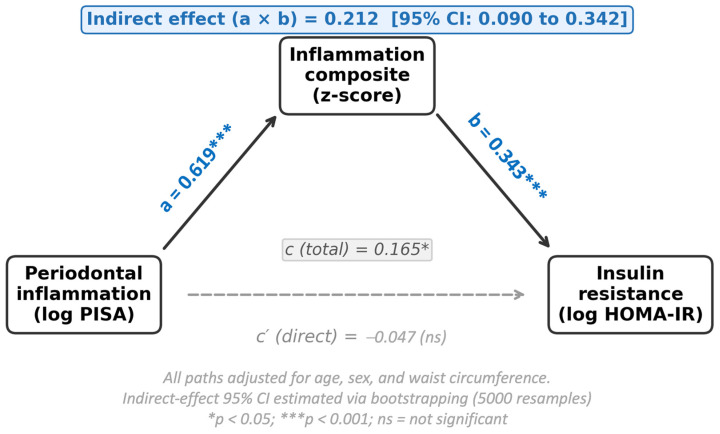
Mediation diagram showing log-scale path coefficients from the model adjusted for age, sex, and waist circumference. The diagram depicts the association of log-transformed PISA with the inflammation composite (path a), the association of the inflammation composite with log-transformed HOMA-IR (path b), and the direct effect of log-transformed PISA on log-transformed HOMA-IR after accounting for the mediator (path c′). Indirect-effect confidence intervals were estimated using bootstrapping (5000 resamples).

When the inflammation composite was included as a mediator, the direct effect of PISA on HOMA-IR (c′) was small and not statistically supported (c′ = −0.047; 95% CI −0.207 to 0.125), whereas the indirect effect (a × b) was positive and statistically supported (a × b = 0.212; 95% CI 0.094 to 0.337), corresponding to an approximate 23.7% increase in HOMA-IR attributable to the inflammation composite within this statistical model. These results are consistent with, but do not establish, a role for systemic inflammation as an intermediate in the relationship between periodontal inflammatory burden and insulin resistance (Table 6; Figure 5). Because exposure, mediator, and outcome were all measured concurrently, temporal ordering cannot be inferred, causality cannot be established, and unmeasured confounding of the mediator–outcome relationship cannot be excluded. These findings should be interpreted as exploratory and hypothesis-generating.

## 4. Discussion

In this cohort of never-smokers attending a periodontal clinic, higher periodontal inflammatory burden was associated with a less favorable metabolic and inflammatory profile. Unadjusted analyses showed that both insulin resistance and systemic inflammation increased across PISA tertiles, with median HOMA-IR rising from 2.01 to 3.17 and median hs-CRP from 0.73 to 2.48 mg/L from the lowest to the highest tertile.

These trends align with current views of periodontitis as an inflammatory condition that may elevate systemic inflammation, thereby affecting insulin signaling and cardiometabolic risk [27,28,29].

In the prespecified primary adjusted model, PISA remained robustly associated with hs-CRP, whereas the association with HOMA-IR was weaker and did not reach conventional statistical significance. Sensitivity analyses, additionally adjusting for BMI or SBP, yielded similar estimates, while inclusion of plaque (% sites) substantially attenuated the associations. This pattern is compatible with plaque being a proximal determinant of periodontal inflammation and a potential source of overadjustment when estimating the total association between periodontal inflammatory burden and systemic outcomes. This is consistent with earlier research linking PISA to CRP and supporting dose–response relationships observed in cross-sectional studies [14,15,30,31,32,33].

The association between PISA and HOMA-IR appeared more model-dependent than the association with hs-CRP. This is biologically plausible because adiposity is strongly associated with insulin resistance and systemic inflammation [34,35] and also with periodontitis risk [36,37,38,39]. Accordingly, shared risk-factor structure may partly explain attenuation after covariate adjustment. The spline analysis also suggested that the PISA–HOMA-IR association may not be strictly linear, which may have contributed to the observed model sensitivity. Additionally, the pattern may partly reflect model specifics: variables such as plaque levels are direct causes of gingival inflammation [40] and may lie on the causal pathway, so including them can lead to overadjustment when estimating the total association between periodontal inflammation and systemic outcomes. This is especially relevant when PISA measures active inflammatory wound surfaces rather than a categorical indicator of disease “severity”.

The exploratory mediation analysis was consistent with systemic inflammation as a possible statistical intermediate in the relationship between periodontal inflammatory burden and insulin resistance, but this finding must be interpreted with considerable caution. Because exposure, mediator, and outcome were measured concurrently, temporal ordering cannot be established, and causality cannot be inferred. Cross-sectional mediation analysis is additionally susceptible to unmeasured confounding of the mediator–outcome relationship, which could bias indirect effect estimates in either direction. The results should therefore be regarded as hypothesis-generating—providing a statistical description consistent with an inflammatory pathway—rather than as mechanistic or causal evidence of mediation.

This pattern is consistent with mechanistic models proposing that periodontal inflammation may promote the release of systemic inflammatory mediators and innate immune activation, which could, in turn, influence insulin signaling and glucose regulation [7,9,41]. The observed association between periodontal inflammation and insulin resistance measures, including HOMA-IR, aligns with recent observational research, though effect sizes and robustness vary by population characteristics, periodontal phenotype, and confounder control [12,19].

From a translational perspective, the present findings indicate that higher periodontal inflammatory burden is associated with an adverse cardiometabolic profile, but several considerations caution against direct clinical inference. This study was not designed to evaluate the utility of PISA as a screening tool; no screening accuracy metrics, referral outcomes, or comparisons with established risk scores were assessed. The specialist periodontal clinic setting, where disease severity is likely higher than in primary dental care or community practice, further limits translation. Whether PISA provides clinically meaningful information beyond simpler periodontal measures routinely available in clinical records—and whether such associations hold in lower-burden settings—remains to be established in dedicated prospective studies.

The clustering of higher PISA with elevated hs-CRP and increased HOMA-IR in the highest tertile is descriptively consistent with prior observations of co-occurring periodontal and metabolic risk in clinical populations [22,23,42]. However, this study was not designed to assess the feasibility or accuracy of cardiometabolic screening in a dental setting, and no screening, referral, or follow-up outcomes were evaluated. While diabetes risk models for dental settings have been developed and partially validated elsewhere [43,44], the present findings do not directly inform their performance or expand their evidence base and should not be interpreted as doing so.

The present research has several strengths, including a focus solely on never-smokers, which reduced confounding by tobacco use and minimized smoking-related distortions in periodontal inflammation measurements, such as suppressed bleeding on probing. The study also enhanced its analysis by quantifying periodontal inflammatory burden with PISA and examining it alongside metabolic and inflammatory biomarkers. Additionally, the sequential modeling approach—ranging from unadjusted to fully adjusted models—provided clear insights into how effect sizes changed with each level of covariate control. The mediation analysis provided a structured framework for investigating systemic inflammation as a potential link between periodontal inflammation and insulin resistance.

Several limitations should be recognized. The cross-sectional design precluded establishing a timeline, and reverse causation remains possible because dysglycemia and insulin resistance might worsen periodontal inflammation through immune dysfunction and impaired tissue repair [8]. The cross-sectional mediation analysis carries additional inferential constraints that deserve explicit recognition: the temporal ordering of PISA, the inflammation composite, and HOMA-IR cannot be determined from these data; unmeasured confounding of the mediator–outcome relationship cannot be excluded; and the term ‘mediation’ reflects a statistical decomposition of observed associations rather than evidence of a causal mechanism. For these reasons, the mediation findings are reported as exploratory and should be confirmed in longitudinal studies with repeated measurements.

Residual confounding by diet, detailed physical activity, psychosocial stress, sleep, and unmeasured socioeconomic factors could not be ruled out. hs-CRP is non-specific and may reflect inflammation unrelated to periodontal disease, and measuring it at a single time point introduces random variability. Although HOMA-IR is a validated marker of insulin resistance, it does not substitute for dynamic testing or clamp-based measures. Moreover, participants were recruited exclusively from a specialist periodontal clinic. The distribution of periodontitis stages—including 28.6% in Stage IV—and the relatively high median PISA values across all tertiles indicate a referred, high-burden population that is not representative of adults with periodontitis in primary dental care or community settings. Associations of the magnitudes observed here may be attenuated or absent in populations with milder disease, substantially curtailing the generalizability of these findings. Any inference about the clinical utility of PISA for cardiometabolic risk identification should therefore be made with considerable caution, and validation in broader, more representative populations is a necessary prerequisite before clinical implications can be drawn.

The inflammation composite used equal weighting of three markers after z-scoring; while PCA confirmed this approach was empirically justified in the present sample, alternative weighting schemes or composite structures might yield different results in other populations or with a different marker panel.

A further structural limitation is the absence of clearly defined comparison groups. The inclusion of a group of individuals with confirmed diabetes but without significant periodontal disease, and of a periodontitis-free age-matched group without metabolic disturbance, would have provided positive and negative reference points against which the observed associations could be more precisely contextualized. Without such controls, it is not possible to determine whether the metabolic differences observed across PISA tertiles reflect a periodontitis-specific effect or a more general gradient associated with inflammatory burden. Future comparative studies incorporating these control arms alongside standardized measurement protocols would substantially strengthen causal interpretation.

Future research should prioritize longitudinal studies that include repeated PISA measurements and serial assessments of inflammatory and metabolic markers. This approach will help clarify causality and distinguish between confounding factors, mediators, and potential bidirectional influences. Using more detailed metabolic indicators—such as OGTT-derived measures, triglyceride–glucose surrogates, or continuous glucose monitoring—may enhance early detection of dysregulation in periodontal populations. Incorporating mechanistic studies with oral microbiome profiling and innate immune activation markers could further elucidate whether specific dysbiotic patterns or inflammatory pathways account for variability in periodontal–metabolic interactions. Before clinical implementation can be considered, research should first establish whether associations between PISA and cardiometabolic markers are replicated in community dental and primary care settings with lower disease burden. If confirmed in such settings, interdisciplinary studies could then assess the incremental value of periodontal inflammation metrics within validated dental diabetes risk frameworks, with attention to screening accuracy, referral rates, and equity in follow-up.

More broadly, the field would benefit from adopting standardized data-collection frameworks that include well-defined positive controls—such as individuals with confirmed diabetes and minimal periodontal disease—and negative controls—such as periodontally and metabolically healthy, age-matched individuals. Such universal reference parameters would reduce between-study heterogeneity, improve the comparability and interpretability of findings across populations and settings, and provide clearer clinical insights into the true nature and magnitude of periodontal–metabolic associations.

## 5. Conclusions

In never-smokers attending a periodontal clinic, higher periodontal inflammatory burden was strongly associated with higher systemic inflammation and showed weaker, model-dependent associations with insulin resistance. The findings are consistent with, but do not prove, an inflammation-linked pathway connecting periodontal inflammation and insulin resistance.

As these findings derive from a specialist periodontal clinic population with a high inflammatory burden, they should not be extrapolated directly to broader dental or community settings. Prospective studies in more representative populations are needed to determine whether quantifying periodontal inflammatory burden adds clinically meaningful information for cardiometabolic risk stratification beyond established indicators.

## Figures and Tables

**Table 1:** Demographic characteristics by PISA tertile.

Variable	Tertile 1 (PISA 47–294 mm^2^)	Tertile 2 (PISA 304–710 mm^2^)	Tertile 3 (PISA 721–3500 mm^2^)	*p*-Value
Participants (n)	51	51	52	
Age (years)	46.2 (12.5)	47.3 (11.9)	52.1 (12.0)	0.035
Sex (Female)	54.9%	56.9%	69.2%	0.272
BMI (kg/m^2^)	26.9 (4.0)	26.3 (3.8)	28.7 (4.3)	0.010
Waist circumference (cm)	89.8 (16.1)	93.5 (13.4)	99.5 (12.4)	0.002
Systolic BP (mmHg)	121.7 (16.1)	125.8 (16.3)	126.3 (20.2)	0.351
Diastolic BP (mmHg)	76.3 (11.9)	77.7 (10.7)	81.1 (10.9)	0.083
Family history of diabetes (Yes)	37.3%	47.1%	32.7%	0.312
Physical activity	L 2.0%, M 64.7%, H 33.3%	L 7.8%, M 72.5%, H 19.6%	L 11.5%, M 73.1%, H 15.4%	0.105
Statin use (Yes)	25.5%	19.6%	25.0%	0.738
Antihypertensive medication use (Yes)	43.1%	31.4%	23.1%	0.092
Systemic corticosteroid use (Yes)	11.8%	3.9%	7.7%	0.335

Data are presented as mean (SD) for continuous variables and n (%) for categorical variables. BP = blood pressure.

**Table 2:** Periodontal characteristics by PISA tertile.

Variable	Tertile 1 (PISA 47–294 mm^2^)	Tertile 2 (PISA 304–710 mm^2^)	Tertile 3 (PISA 721–3500 mm^2^)	*p*-Value
Number of teeth	27.4 (1.2)	26.5 (1.8)	24.3 (2.1)	<0.001
Mean PPD (mm)	2.8 (0.7)	3.2 (0.7)	3.9 (0.8)	<0.001
Mean CAL (mm)	1.6 (1.1)	2.1 (1.0)	3.2 (1.2)	<0.001
BOP (% sites)	26.6 (16.4)	44.0 (18.0)	68.2 (16.4)	<0.001
Plaque (% sites)	24.9 (15.7)	38.8 (15.6)	62.9 (16.8)	<0.001
PISA (mm^2^)	225.00 [157.00, 264.50]	511.00 [406.00, 600.00]	1092.50 [840.50, 1432.75]	<0.001

Data are presented as mean (SD), except PISA, which is presented as median [IQR]. PPD: periodontal probing depth; CAL: clinical attachment loss; BOP: bleeding on probing; PISA: periodontal inflamed surface area.

**Table 3:** Metabolic biomarkers by PISA tertile.

Variable	Tertile 1 (PISA 47–294 mm^2^)	Tertile 2 (PISA 304–710 mm^2^)	Tertile 3 (PISA 721–3500 mm^2^)	*p*-Value
Fasting glucose (mmol/L)	5.30 [4.66, 5.70]	5.21 [4.67, 5.81]	5.68 [5.22, 5.99]	0.018
Fasting insulin (µIU/mL)	8.33 [6.46, 15.48]	10.19 [6.78, 13.56]	14.20 [9.67, 20.58]	<0.001
HbA1c (%)	5.33 [5.09, 5.77]	5.38 [5.04, 5.81]	5.60 [5.32, 5.88]	0.063
HOMA-IR	2.01 [1.31, 3.82]	2.27 [1.48, 3.57]	3.17 [2.43, 5.63]	<0.001
hs-CRP (mg/L)	0.73 [0.48, 1.01]	1.02 [0.58, 2.00]	2.48 [1.36, 4.35]	<0.001
IL-6 (pg/mL)	1.26 [0.80, 1.79]	1.98 [1.22, 2.73]	2.08 [1.67, 4.00]	<0.001
TNF-α (pg/mL)	1.68 [1.31, 2.35]	2.17 [1.54, 3.14]	3.29 [2.38, 5.33]	<0.001

Data are presented as median [IQR]. *p*-values are from across-tertile comparisons.

**Table 4:** Periodontitis stage and grade distribution (N = 154).

	n	%
Stage		
I	43	27.9
II	36	23.4
III	31	20.1
IV	44	28.6
Grade		
A	64	41.6
B	56	36.4
C	34	22.1

**Table 5:** Association of periodontal inflammatory burden with HOMA-IR and hs-CRP in primary and sensitivity models.

Outcome	Model	% Change per IQR log(PISA)	95% CI	*p*-Value	Adj. R^2^
HOMA-IR	Crude	39.9%	20.8% to 62.1%	<0.001	0.103
HOMA-IR	Primary adjusted	15.7%	−0.8% to 34.9%	0.064	0.279
HOMA-IR	Primary adjusted + BMI	15.1%	−0.8% to 33.6%	0.063	0.305
HOMA-IR	Primary adjusted + SBP	17.4%	1.3% to 36.1%	0.034	0.323
HOMA-IR	Primary adjusted + plaque	1.0%	−18.0% to 24.3%	0.925	0.292
HOMA-IR	Primary adjusted + plaque + BMI	2.9%	−16.1% to 26.3%	0.782	0.312
hs-CRP	Crude	103.2%	73.8% to 137.6%	<0.001	0.297
hs-CRP	Primary adjusted	85.2%	53.1% to 124.1%	<0.001	0.312
hs-CRP	Primary adjusted + BMI	83.9%	52.5% to 121.8%	<0.001	0.355
hs-CRP	Primary adjusted + SBP	85.8%	52.9% to 125.8%	<0.001	0.308
hs-CRP	Primary adjusted + plaque	15.2%	−8.7% to 45.5%	0.234	0.447
hs-CRP	Primary adjusted + plaque + BMI	18.2%	−6.1% to 48.8%	0.154	0.471

Models report percent change in the outcome per interquartile range (IQR) increase in log-transformed PISA. The primary adjusted model includes age, sex, waist circumference, education, physical activity category, family history of diabetes, and medication use (statins, antihypertensives, systemic corticosteroids).

**Table 6:** Mediation analysis for the association between PISA and HOMA-IR via the inflammation composite (adjusted for age, sex, and waist circumference).

Effect	Estimate (Log Scale)	95% CI (Log Scale)	Approximate % Change in HOMA-IR
Total effect (c)	0.165	0.021 to 0.314	18.0%
Direct effect (c′)	−0.047	−0.207 to 0.125	−4.6%
Indirect effect (a × b)	0.212	0.094 to 0.337	23.7%

## Data Availability

The deidentified clinical, periodontal, metabolic, and inflammatory biomarker data supporting the findings of this study are available from the corresponding author upon reasonable request. Requests will be assessed in accordance with the institutional ethics approval, participant consent, and applicable data protection regulations. Laboratory output files and biomarker records are retained on institutional password-protected systems. No publicly accessible dataset was generated or deposited because the data contain participant-level clinical and biomarker information.

## Abbreviations

The following abbreviations are used in this manuscript:

AAP/EFP	American Academy of Periodontology/European Federation of Periodontology
ANOVA	analysis of variance
BMI	body mass index
BOP	bleeding on probing
BP	blood pressure
CAL	clinical attachment level/loss
CC BY	Creative Commons Attribution license
CI	confidence interval
DBP	diastolic blood pressure
ECLIA	electrochemiluminescence immunoassay
EDTA	ethylenediaminetetraacetic acid
ELISA	enzyme-linked immunosorbent assay
HbA1c	glycated hemoglobin
HC3	heteroskedasticity-consistent type 3 standard errors
HOMA-IR	Homeostasis Model Assessment of Insulin Resistance
HPLC	high-performance liquid chromatography
hs-CRP	high-sensitivity C-reactive protein
ICC	intraclass correlation coefficient
IFCC	International Federation of Clinical Chemistry and Laboratory Medicine
IL-6	interleukin-6
IQR	interquartile range
NGSP	National Glycohemoglobin Standardization Program
OGTT	oral glucose tolerance test
PISA	periodontal inflamed surface area
PPD	probing pocket depth
R	R Foundation for Statistical Computing statistical software
SBP	systolic blood pressure
SD	standard deviation
STROBE	Strengthening the Reporting of Observational Studies in Epidemiology
TNF-α	tumor necrosis factor-alpha
VIF	variance inflation factor

## References

[1] Łasica A., Golec P., Laskus A., Zalewska M., Gędaj M., Popowska M. (2024). Periodontitis: Etiology, Conventional Treatments, and Emerging Bacteriophage and Predatory Bacteria Therapies. Front. Microbiol..

[2] Foroughi M., Torabinejad M., Angelov N., Ojcius D.M., Parang K., Ravnan M., Lam J. (2025). Bridging Oral and Systemic Health: Exploring Pathogenesis, Biomarkers, and Diagnostic Innovations in Periodontal Disease. Infection.

[3] Martínez-García M., Hernández-Lemus E. (2021). Periodontal Inflammation and Systemic Diseases: An Overview. Front. Physiol..

[4] Tattar R., da Costa B.D.C., Neves V.C.M. (2025). The Interrelationship between Periodontal Disease and Systemic Health. Br. Dent. J..

[5] Villoria G.E.M., Fischer R.G., Tinoco E.M.B., Meyle J., Loos B.G. (2024). Periodontal Disease: A Systemic Condition. Periodontology.

[6] Sufaru I.-G., Teslaru S., Pasarin L., Iovan G., Stoleriu S., Solomon S.M. (2022). Host Response Modulation Therapy in the Diabetes Mellitus–Periodontitis Conjuncture: A Narrative Review. Pharmaceutics.

[7] Graves D.T., Levine M.A., Aldosary S., Demmer R.T. (2026). Understanding the Periodontitis–Diabetes Linkage: Mechanisms and Evidence. J. Dent. Res..

[8] Nguyen T.T., Bandeira M., Giannopoulou C., Zekeridou A., Ryu D., Gariani K. (2026). Periodontitis and Diabetes: A Bidirectional Link. Acta Diabetol..

[9] Sohn Y., Jeong H.J., Park J. (2026). The Link between Hyperinsulinemia and Periodontitis in Diabetics. J. Dent. Res..

[10] Xiang D.-D., Sun Y.-X., Jiao C., Guo Y.-Q., Fei Y.-X., Ren B.-Q., He X.-T., Li X. (2025). Diabetes and Periodontitis: The Role of a High-Glucose Microenvironment in Periodontal Tissue Cells and Corresponding Therapeutic Strategies. Stem Cell Res. Ther..

[11] El Chaar E. (2025). Periodontal Disease: A Contributing Factor to Adverse Outcome in Diabetes. J. Diabetes.

[12] Lang Y., Song X., Chen Y., Mei H., Wu C., Zhang R., Xue C. (2025). Association between the Indicators of Insulin Resistance and Periodontitis: A Study Using Data from the National Health and Nutrition Examination Survey 2009–2014. BMC Oral Health.

[13] Nomura Y., Morozumi T., Numabe Y., Ogata Y., Nakayama Y., Sugaya T., Nakamura T., Sato S., Takashiba S., Sekino S. (2021). Estimation of the Periodontal Inflamed Surface Area by Simple Oral Examination. J. Clin. Med..

[14] Kumari R., Banerjee A., Verma A., Kumar A., Biswas N., Kumari P. (2024). Assessing the Correlation of Periodontal Inflamed Surface Area (PISA) with Systemic Inflammatory Markers. Cureus.

[15] Yan Y., Sharma P., Suvan J., D’Aiuto F. (2025). The Association of Periodontal Inflammation and Systemic Health Indicators: A Machine Learning Approach. J. Clin. Periodontol..

[16] Li M., Chi X., Wang Y., Setrerrahmane S., Xie W., Xu H. (2022). Trends in Insulin Resistance: Insights into Mechanisms and Therapeutic Strategy. Signal Transduct. Target. Ther..

[17] Diniz M.F.H.S., Beleigoli A.M.R., Schmidt M.I., Duncan B.B., Ribeiro A.L.P., Vidigal P.G., Benseñor I.M., Lotufo P.A., Santos I.S., Griep R.H. (2020). Homeostasis Model Assessment of Insulin Resistance (HOMA-IR) and Metabolic Syndrome at Baseline of a Multicentric Brazilian Cohort: ELSA-Brasil Study. Cad. Saude Publica.

[18] Lee J., Kim M.H., Jang J.Y., Oh C.M. (2023). Assessment HOMA as a Predictor for New Onset Diabetes Mellitus and Diabetic Complications in Non-Diabetic Adults: A KoGES Prospective Cohort Study. Clin. Diabetes Endocrinol..

[19] Caleb C.L., Dharuman S., Sundhar M.P., Chellapandi S., Balaji U.G. (2025). Evaluation of the Association between Insulin Resistance and the Development of Periodontitis in Individuals with Varying Levels of Dietary Sugar Intake—A Pilot Study. J. Indian Soc. Periodontol..

[20] Mouliou D.S. (2023). C-Reactive Protein: Pathophysiology, Diagnosis, False Test Results and a Novel Diagnostic Algorithm for Clinicians. Diseases.

[21] Machado V., Botelho J., Escalda C., Hussain S.B., Luthra S., Mascarenhas P., Orlandi M., Mendes J.J., D’Aiuto F. (2021). Serum C-Reactive Protein and Periodontitis: A Systematic Review and Meta-Analysis. Front. Immunol..

[22] Laniado N., Cloidt M.A., Badner V.C.M. (2021). Chairside Diabetes Screening: A Survey of Dental Providers at the Largest Municipal Healthcare System in the United States. Oral Health Prev. Dent..

[23] Mohd Norwir N.A., Mohd-Said S., Abdul Aziz A.F., Mohd-Dom T.N. (2025). Leveraging Dental Visits for Systemic Health: Diabetes Screening and Referral Compliance in Periodontitis Patients in Malaysia. J. Clin. Med..

[24] Ainamo J., Bay I. (1975). Problems and Proposals for Recording Gingivitis and Plaque. Int. Dent. J..

[25] Nesse W., Abbas F., van der Ploeg I., Spijkervet F.K., Dijkstra P.U., Vissink A. (2008). Periodontal Inflamed Surface Area: Quantifying Inflammatory Burden. J. Clin. Periodontol..

[26] Tonetti M.S., Greenwell H., Kornman K.S. (2018). Staging and Grading of Periodontitis: Framework and Proposal of a New Classification and Case Definition. J. Periodontol..

[27] Pirih F.Q., Monajemzadeh S., Singh N., Sinacola R.S., Shin J.M., Chen T., Fenno J.C., Kamarajan P., Rickard A.H., Travan S. (2021). Association between Metabolic Syndrome and Periodontitis: The Role of Lipids, Inflammatory Cytokines, Altered Host Response, and the Microbiome. Periodontol..

[28] Aizenbud I., Wilensky A., Almoznino G. (2023). Periodontal Disease and Its Association with Metabolic Syndrome—A Comprehensive Review. Int. J. Mol. Sci..

[29] Kim M.Y., Pang E.K. (2025). Relationship between Periodontitis and Systemic Health Conditions: A Narrative Review. Ewha Med. J..

[30] Botelho J., Machado V., Leira Y., Proença L., Mendes J.J. (2021). Periodontal Inflamed Surface Area Mediates the Link between Homocysteine and Blood Pressure. Biomolecules.

[31] Miki K., Kitamura M., Hatta K., Kamide K., Gondo Y., Yamashita M., Takedachi M., Nozaki T., Fujihara C., Kashiwagi Y. (2021). Periodontal Inflamed Surface Area Is Associated with hs-CRP in Septuagenarian Japanese Adults in Cross-Sectional Findings from the SONIC Study. Sci. Rep..

[32] Onabanjo O.A., Nwhator S.O., Arogundade F.A. (2023). Association between periodontal inflamed surface area and systemic inflammatory biomarkers among pre-dialysis chronic kidney disease patients. Niger. Postgrad. Med. J..

[33] Mance Kristan R., Jurgec S., Potočnik U., Marhl M., Gašperšič R. (2024). The Association between Periodontal Inflamed Surface Area (PISA), Inflammatory Biomarkers, and Mitochondrial DNA Copy Number. J. Clin. Med..

[34] Zhu B., Guo X., Xu H., Jiang B., Li H., Wang Y., Yin Q., Zhou T., Cai J.J., Glaser S. (2021). Adipose Tissue Inflammation and Systemic Insulin Resistance in Mice with Diet-Induced Obesity Is Possibly Associated with Disruption of PFKFB3 in Hematopoietic Cells. Lab. Investig..

[35] Hughes M.J., McGettrick H.M., Sapey E. (2020). Shared Mechanisms of Multimorbidity in COPD, Atherosclerosis and Type-2 Diabetes: The Neutrophil as a Potential Inflammatory Target. Eur. Respir. Rev..

[36] Maulani C., Auerkari E.I., Masulili S.L.C., Kusdhany L.S., Prahasanti C., Soedarsono N. (2021). Obesity Correlated to a Higher Risk of Acquiring Periodontitis: A Cross-Sectional Study. F1000Research.

[37] Iwashita M., Hayashi M., Nishimura Y., Yamashita A. (2021). The Link between Periodontal Inflammation and Obesity. Curr. Oral Health Rep..

[38] Reytor-González C., Parise-Vasco J.M., González N., Simancas-Racines A., Zambrano-Villacres R., Zambrano A.K., Simancas-Racines D. (2024). Obesity and Periodontitis: A Comprehensive Review of Their Interconnected Pathophysiology and Clinical Implications. Front. Nutr..

[39] Esperouz F., Ciavarella D., Di Gioia C., Serviddio G., Lorusso M., Lo Russo L. (2026). Is Obesity a Risk Factor for Periodontitis? A Systematic Review and Meta-Analysis. Obes. Rev..

[40] Reiniger A.P.P., Maier J., Wikesjö U.M.E., Moreira C.H.C., Kantorski K.Z. (2021). Correlation between dental plaque accumulation and gingival health in periodontal maintenance patients using short or extended personal oral hygiene intervals. J. Clin. Periodontol..

[41] Muñoz-Carrillo J.L., Palomeque-Molina P.I., Villacis-Valencia M.S., Gutiérrez-Coronado O., Chávez-Ruvalcaba F., Vázquez-Alcaraz S.J., Villalobos-Gutiérrez P.T., Palomeque-Molina J. (2025). Relationship between periodontitis, type 2 diabetes mellitus and COVID-19 disease: A narrative review. Front. Cell. Infect. Microbiol..

[42] Glurich I., Bartkowiak B., Berg R.L., Acharya A. (2018). Screening for dysglycaemia in dental primary care practice settings: Systematic review of the evidence. Int. Dent. J..

[43] Chee H.K., Abbas F., van Winkelhoff A.J., Tjakkes G.H., Htoon H.M., Li H., de Waal Y., Vissink A., Seneviratne C.J. (2025). Identifying Undiagnosed Diabetes and Prediabetes in the Dental Setting in an Asian Population—A Clinical Risk Model. J. Clin. Periodontol..

[44] Rodrigues C., Machado V., Proença L., Mendes J.J., Kocher T., Holtfreter B., Yonel Z., Botelho J. (2026). Validation of the Diabetes Risk Assessment in Dentistry Score in NHANES 2009–2014. J. Clin. Periodontol..

